# Wilms' Tumor Protein Induces an Epithelial-Mesenchymal Hybrid Differentiation State in Clear Cell Renal Cell Carcinoma

**DOI:** 10.1371/journal.pone.0102041

**Published:** 2014-07-15

**Authors:** Valerie B. Sampson, Justin M. David, Isabel Puig, Pratima U. Patil, Antonio García de Herreros, George V. Thomas, Ayyappan K. Rajasekaran

**Affiliations:** 1 Nemours Center for Childhood Cancer Research, Alfred I. duPont Hospital for Children, Wilmington, Delaware, United States of America; 2 Department of Biological Sciences, University of Delaware, Newark, Delaware, United States of America; 3 IMIM (Institut Hospital del Mar d'Investigacions Mèdiques), Barcelona, Spain; 4 Knight Cancer Institute, Oregon Health and Sciences University, Portland, Oregon, United States of America; University of British Columbia, Canada

## Abstract

The Wilms' tumor transcription factor (WT1) was originally classified as a tumor suppressor, but it is now known to also be associated with cancer progression and poor prognosis in several malignancies. WT1 plays an essential role in orchestrating a developmental process known as mesenchymal-to-epithelial transition (MET) during kidney development, but also induces the reverse process, epithelial-to-mesenchymal transition (EMT) during heart development. WT1 is not expressed in the adult kidney, but shows elevated expression in clear cell renal cell carcinoma (ccRCC). However, the role of WT1 in this disease has not been characterized. In this study, we demonstrate that WT1 is upregulated in ccRCC cells that are deficient in the expression of the von Hippel-Lindau tumor suppressor protein (VHL). We found that WT1 transcriptionally activated Snail, a master transcriptional repressor that is known to induce EMT. Although Snail represses E-cadherin and induces mesenchymal characteristics, we found partial maintenance of E-cadherin and associated epithelial characteristics in kidney cells and ccRCC cells that express WT1, since WT1 upregulates E-cadherin expression and competes with Snail repression. These findings support a novel paradigm in which WT1 induces an epithelial-mesenchymal hybrid transition (EMHT), characterized by Snail up-regulation with E-cadherin maintenance, a tumor cell differentiation state in which cancer cells keep both EMT and MET characteristics which may promote tumor cell plasticity and tumor progression.

## Introduction

Renal cell carcinomas (RCCs) are a heterogeneous group of deadly and treatment-resistant malignancies. The predominant subtype of RCC is clear cell RCC (ccRCC) and is characterized by loss-of-function mutations of the von Hippel-Lindau (*VHL*) tumor suppressor gene [1]. *VHL* encodes a protein of the same name that is a direct oxygen-dependent negative regulator of the α-subunits of the transcription factor hypoxia-inducible factor (HIF-α). In normoxic conditions, VHL binds to HIF-α and promotes its ubiquitylation and subsequent degradation by the proteasome [1]. However, hypoxia prevents VHL binding which stabilizes HIF-α and permits the activation of target genes that regulate cellular adaptation to low oxygen [1]. Loss of VHL in ccRCC abrogates oxygen-dependent regulation of HIF resulting in aberrant chronic activation of HIF regardless of cellular oxygenation. The HIF transcriptional program governs many diverse processes that facilitate cancer progression, including angiogenesis, metabolism, proliferation, survival, and metastasis [2].

The Wilms' tumor gene (*WT1*) was originally discovered as a tumor suppressor gene inactivated in a subset (∼15%) of pediatric renal cancers unrelated to RCC known as Wilms' tumors [3]. This cancer arises when pluripotent progenitor cells of the metanephric mesenchyme fail to undergo differentiation into the glomeruli and tubules of the nephron [4]. This process, termed mesenchymal-to-epithelial transition (MET), is activated by the -KTS splice variant of the *WT1* gene, which serves as a transcription factor [3]. Conversely, in heart development WT1 has been shown to activate the reverse process, epithelial-to-mesenchymal transition (EMT), in the epicardial cells that generate the cardiovascular progenitor cells which then differentiate into various adult cardiac cells (coronary smooth muscle, interstitial fibroblasts, cardiomyocytes) [5]. In addition, the highest levels of WT1 expression in adults are found in the podocytes (kidney), Sertoli cells (testis), and mesothelial cells, and all of these cell types share the capacity to readily switch between epithelial and mesenchymal phenotypes [6], [7]. These observations suggest that WT1 mediates reciprocal transitions between these phenotypes.

Transitions in cellular differentiation between epithelial and mesenchymal states are critical not only in organ development and wound healing, but also appear to be co-opted during cancer progression. Epithelial cells are typically immobilized within tightly bound layers and exhibit apical-basolateral plasma membrane polarity and extensive cell-cell and cell-matrix adhesions. Vital to epithelial cells is E-cadherin, a Ca^2+^-dependent cell-cell adhesion molecule that forms the core of the adherens junctions that physically links cells together in close proximity to promote the well-differentiated epithelial phenotype [8]–[10]. In contrast to epithelial cells, mesenchymal cells exhibit an elongated and asymmetric morphology, and form only transient adhesions with neighboring cells [11]. This phenotype promotes the dissolution of tissue integrity and enhances cell motility and invasion [12], [13]. Loss of E-cadherin expression is a crucial event in the establishment of the mesenchymal phenotype, and transcriptional repressors such as Snail downregulate E-cadherin during EMT [14]–[16].

EMT is thought to occur during the progression of cancer to metastatic disease. This not only confers invasive properties, but also endows tumor cells with stem cell-like characteristics such as self-renewal and therapy-resistance [17]. However, clinical observations have revealed that metastases derived from a variety of carcinoma types often display overtly epithelial differentiation [18]. Recent evidence suggests that disseminated tumor cells may undergo MET in order to better facilitate colonization (i.e. proliferation) at the foreign site [17], [19]. Although tumor cell differentiation appears to be highly plastic, the cellular and molecular regulators of this phenotypic plasticity are not well known. WT1 expression has been reported to be upregulated in a variety of solid tumors, including in ccRCC where it seems to act as an oncogene [7], [20], yet little is known about the pathophysiological consequences of WT1 expression in cancer. In this study, we report that WT1 expression is increased in VHL-deficient ccRCC cells. We show that WT1 directly upregulates Snail while also promoting the expression of E-cadherin. Further, WT1-expressing renal cells exhibited epithelial-like morphological features and epithelial junctions, even in the presence of Snail, while simultaneously also expressing various EMT and MET markers. Our results indicate that WT1 induces features of both EMT and MET in ccRCC and suggest that it may regulate phenotypic plasticity by promoting an epithelial-mesenchymal hybrid differentiation state in cancer.

## Results

### Knockdown of VHL increases WT1 expression

**Figure 1:**
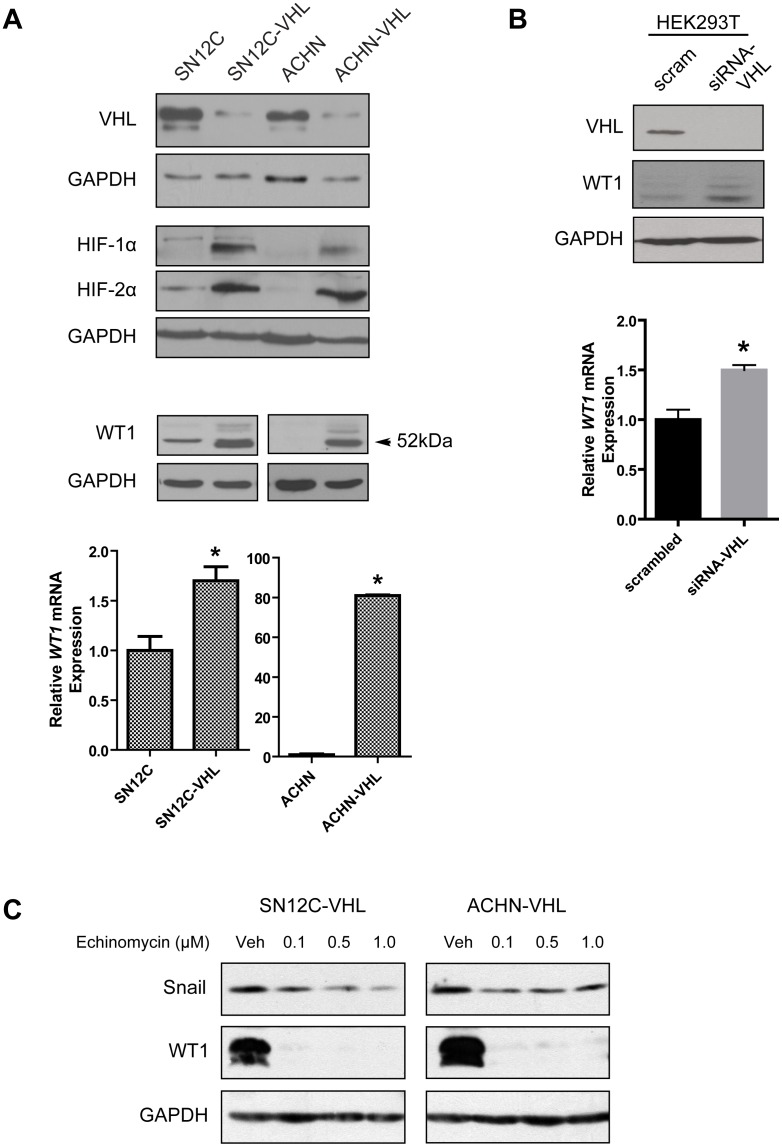
Knockdown of VHL increases WT1 expression. (A) Confirmation of VHL knockdown (top), and analysis of WT1 protein (middle) and mRNA (bottom) in the isogenic SN12C and ACHN cell lines. (B) HEK293T cells were transfected with scrambled oligoneucleotides or VHL-specific siRNAs and WT1 protein (top) and mRNA (bottom) was measured. Graphs show the mean±SD of one representative of three independent experiments. *, *P*<0.05. (C) SN12C-VHL (left) and ACHN-VHL (right) cells were treated with vehicle (DMSO) or echinomycin (0.1, 0.5, 1.0 µM) for 24 hr and protein expression was assessed by immunoblot.

Increased expression of WT1 has been previously reported in ccRCC patient tumor samples [20]. Since functional inactivation of the *VHL* gene is a common defect in ccRCC, we hypothesized that loss of VHL might be associated with increased WT1 expression. To test this hypothesis, we used two well-characterized isogenic pairs of human ccRCC cell lines, one pair expresses wild-type VHL (parental SN12C and ACHN cells), the other pair expresses a shRNA-VHL knockdown construct (SN12C-VHL and ACHN-VHL) [21]. Immunoblot analysis confirmed knockdown of VHL expression with corresponding stabilization of HIF-1α and HIF-2α in these pairs (Fig. 1A, top panel), and also revealed increased WT1 expression in the VHL-deficient cell lines (Fig. 1A, middle panel). Elevated WT1 protein expression corresponded with a significant increase in WT1 transcript levels (Fig. 1A, bottom panel). Consistently, knockdown of endogenous VHL in normal embryonic kidney cells (HEK293T) also resulted in upregulation of WT1 protein and mRNA (Fig. 1B).

Since loss of VHL results in constitutive activation of HIF [22], and WT1 was reported to be a HIF target gene [23], we investigated whether WT1 expression in the VHL-knockdown cells was associated with HIF activity. Using echinomycin, which blocks the binding of HIF-1α/HIF-β and HIF-2α/HIF-β heterodimers to DNA [24], we found that inhibition of HIF activity partially suppressed the expression of Snail, a well-known HIF target gene [25], and totally suppressed WT1 expression in SN12C-VHL and ACHN-VHL cells (Fig. 1C). Altogether, these data indicate that loss of VHL expression induces WT1 in a manner that involves HIF.

### WT1 regulates Snail expression

**Figure 2:**
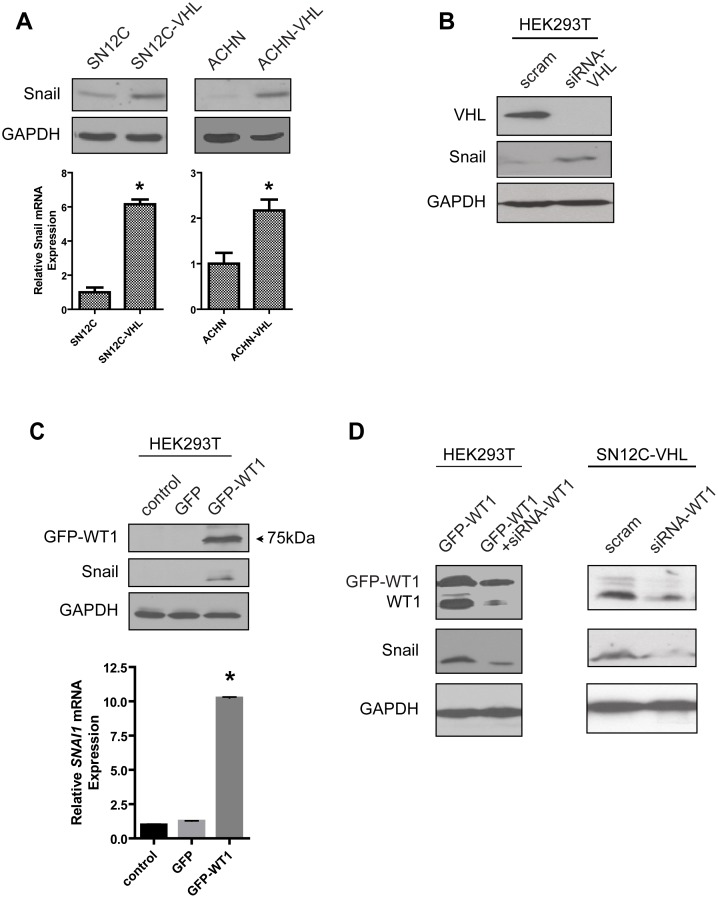
WT1 regulates Snail expression. (A) Analysis of Snail protein (top) and mRNA (bottom) in the isogenic SN12C and ACHN cell lines. (B) HEK293T cells were transfected with scrambled oligonucleotides or VHL-specific siRNAs and Snail protein was measured. (C) HEK293T cells were transfected with GFP or GFP-WT1 and Snail protein (top) and mRNA (bottom) was measured. Graphs show mean±SD of one representative of three independent experiments. *, *P*<0.05. (D) HEK293T and SN12C-VHL cells were transfected as indicated and protein expression was assessed by immunoblot.

During heart development, WT1-expressing cells cross the pericardial cavity and spread over the surface of the myocardium to form the epicardial layer [26]. Subsequently, it has been shown that WT1 expression in epicardiocytes induces Snail, a master regulator of EMT, which is also expressed during carcinoma progression [27]. Consistent with these reports, we observed increased Snail protein and mRNA expression in the VHL-deficient SN12C and ACHN cells compared to the parental cells (Fig. 2A). Transient knockdown of VHL in HEK293T cells also produced a similar induction of Snail protein expression (Fig. 2B). Next, to investigate whether overexpression of WT1 upregulates Snail, we transfected HEK293T cells with a plasmid encoding WT1 fused to GFP. Transient expression of GFP-WT1 resulted in increased Snail protein and mRNA levels (Fig. 2C). In a complementary experiment, siRNA-mediated knockdown of WT1 in the GFP-WT1-overexpressing HEK293T cells was accompanied by the specific loss of Snail expression (Fig. 2D, left). In agreement with these findings, transient knockdown of WT1 in SN12C-VHL cells resulted in dramatic reduction in Snail protein expression (Fig. 2D, right). Taken together, these results demonstrate that in VHL-deficient ccRCC cells, increased WT1 expression is associated with the induction of Snail.

### WT1 transcriptionally upregulates Snail in VHL-knockdown cells

**Figure 3:**
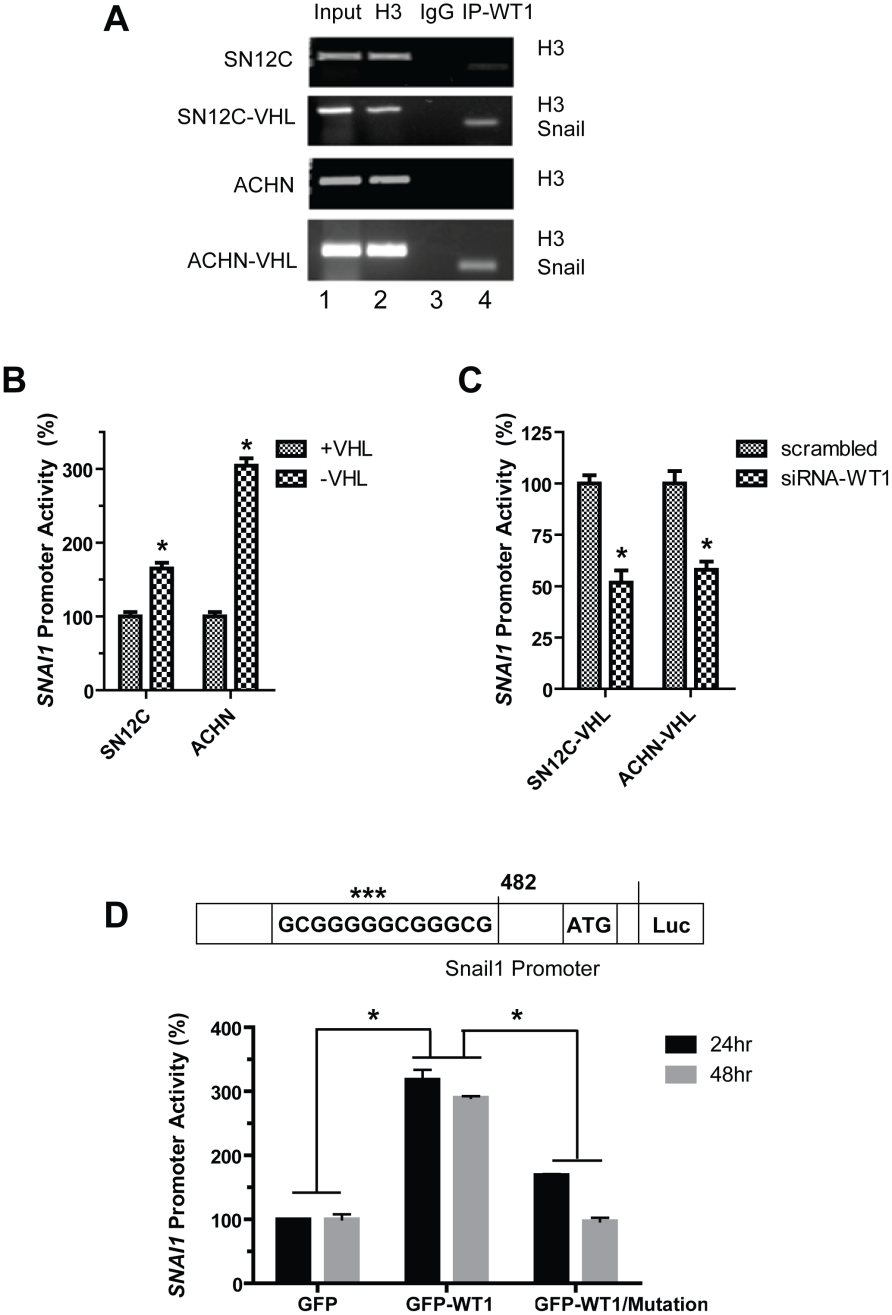
WT1 transcriptionally upregulates Snail in VHL-deficient cells. (A) ChIP assays for WT1 were performed in the isogenic SN12C and ACHN cell lines. WT1 was immunoprecipitated and the bound DNA fragments were analyzed by PCR amplification for Snail. Histone H3 and rabbit IgG were used as positive and negative controls, respectively. Human RPL30 exon3 primers amplified a 160 bp fragment from immunoprecipitation of H3. Snail primers amplified a 120 bp fragment from immunoprecipitation using WT1 antibody. (B) *SNAI1* promoter luciferase activity was measured in the isogenic SN12C and ACHN cells. (C) SN12C-VHL and ACHN-VHL cells were cotransfected with either scrambled or siRNA-WT1 oligonucleotides, and *SNAI1* promoter luciferase activity was measured. Graphs depict mean±SD of one representative of three independent experiments. (D) Top, schematic representation of WT1 binding sequence within the Snail promoter with mutated residues highlighted with asterisks (***). Bottom, HEK293T cells were cotransfected with GFP or GFP-WT1 and either wild-type or mutated *SNAI1* promoter constructs and luciferase activity was measured. Graph depicts mean±SD of three independent experiments. *, *P*<0.05.

The proximal Snail gene (*SNAI1*) promoter region has previously been reported to contain a highly conserved functional WT1 binding sequence, (5′-GCGGGGGCGGGCG-3′) [27]. Since we observed positive regulation of Snail expression by WT1, we tested whether WT1 transcriptionally upregulates *SNAI1* gene expression in ccRCC cells. Chromatin immunoprecipitation (ChIP) experiments were performed using the isogenic ccRCC cell lines to determine whether endogenous WT1 interacts with the *SNAI1* promoter *in vivo*. We found that WT1 binds specifically to the *SNAI1* promoter (Fig. 3A, lane 4), and binding was markedly enhanced in the VHL-knockdown cells. We performed the luciferase reporter assay using a plasmid containing the human *SNAI1* promoter (including the putative WT1-binding motif, nucleotides −495 to −482 with respect to the transcription start site; Fig. 3D, schematic) fused to firefly luciferase [16]. Transfection of this plasmid into the isogenic ccRCC cell lines revealed significantly enhanced *SNAI1* promoter activity in the VHL-knockdown cells by 60% (SN12C-VHL) and 200% (ACHN-VHL) compared to the respective parental cell lines (Fig. 3B). In contrast, siRNA-mediated knockdown of WT1 in these VHL-knockdown cells significantly reduced *SNAI1* promoter activity to by 50% (SN12C-VHL) and 40% (ACHN-VHL) (Fig. 3C). In agreement with our previous data, overexpression of GFP-WT1 in HEK293T cells significantly increased *SNAI1* promoter activity by approximately 3-fold at both 24 and 48 hrs relative to control GFP vector-transfected cells (Fig. 3D, graph). Next, we mutated three nucleotides within the WT1-binding site (Fig. 3D schematic, shown by asterisks) to determine whether WT1 interacts directly with the *SNA1* promoter. Mutation of this sequence resulted in a substantial reduction of promoter activity by approximately 2-fold and 3-fold at 24 and 48 hrs, respectively, in GFP-WT1-expressing cells. These data demonstrate that WT1 directly binds to the *SNAI1* gene promoter and increases its expression in VHL-knockdown ccRCC cells.

### WT1 competes with Snail to regulate E-cadherin expression

**Figure 4:**
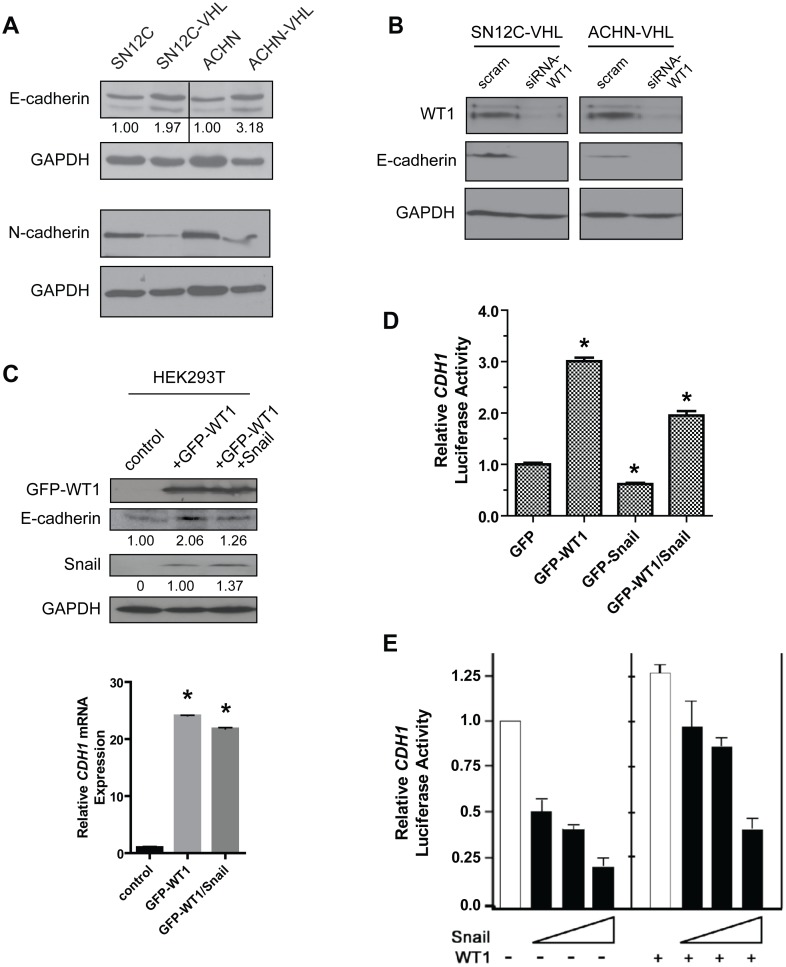
WT1 upregulates E-cadherin expression in the presences of Snail. (A) Immunoblot analysis of E-cadherin and N-cadherin expression in the isogenic SN12C and ACHN cell lines. (B) VHL-knockdown RCC cells were transfected with scrambled or WT1-specifc siRNAs and E-cadherin expression was assessed by immunoblot. (C) Top, HEK293T cells were transfected as indicated and protein expression was assessed by immunoblot. Bottom, analysis of E-cadherin mRNA levels. (D, E) HEK293T (D) or MDCK (E) cells were cotransfected with the indicated plasmids and the *CDH1* promoter reporter construct, and luciferase activity was measured. Graphs represent mean±SD of one representative experiment. *, *P*<0.05.

Snail is well-known to induce EMT through repression of the E-cadherin gene (*CDH1*) [15]. WT1 has also recently been reported to repress *CDH1* [5], [28]. Because VHL-knockdown resulted in enhanced expression of both proteins, we hypothesized that E-cadherin expression would be reduced in these cells. Surprisingly, we found the opposite result; E-cadherin expression was significantly elevated in SN12C-VHL cells and ACHN-VHL cells relative to the respective parental cell lines (Fig. 4A). Additionally, expression of N-cadherin, a marker of mesenchymal differentiation [11], [29] was markedly reduced in VHL-knockdown cells (Fig. 4A). These results suggest that loss of VHL in these ccRCC cell lines may be associated with molecular features of epithelial differentiation despite elevated WT1 and Snail expression.

Although WT1 has been reported to downregulate E-cadherin (5,28), other authors have shown that WT1 directly activates E-cadherin in NIH 3T3 cells [30]. To determine whether WT1 regulates E-cadherin in a similar manner in renal cells, we transfected the VHL-knockdown RCC cells with siRNA-WT1 oligonucleotides. Our results show that loss of WT1 significantly reduced E-cadherin expression (Fig. 4B). In agreement with these findings, we also found that overexpression of GFP-WT1 in HEK293T cells enhanced E-cadherin protein (Fig. 4C, top panel) and mRNA (Fig. 4C, bottom panel) expression. Interestingly, Snail was also induced in these cells (Fig. 4C, top panel). The simultaneous overexpression of both GFP-WT1 and Snail in HEK293T cells produced only a partial reduction in E-cadherin protein (Fig. 4C, top panel) and mRNA (Fig. 4C, bottom panel). In a luciferase assay, using HEK293T cells and the human *CDH1* promoter reporter construct, we found that GFP-WT1 overexpression significantly increased *CDH1* promoter activity by 3-fold whereas Snail overexpression repressed activity ∼40%, as anticipated (Fig. 4D). However, *CDH1* promoter activity was still significantly enhanced (2-fold) with concomitant expression of both GFP-WT1 and Snail (Fig. 4D). We also performed this assay in Madin-Darby canine kidney (MDCK) cells using increasing amounts of exogenous human Snail in the absence or presence of exogenous human WT1. Fig. 4E shows a dose-dependent reduction in *CDH1* promoter activity with increasing concentrations of Snail in the absence of WT1 (left panel). In the presence of WT1, the activity of the *CDH1* promoter also decreased in a dose-dependent manner with increasing Snail, however this decrease was less pronounced with increasing concentrations of WT1 (right panel). Collectively, our results demonstrate that WT1 positively regulates both E-cadherin and Snail in renal cells, and partially protects against Snail-mediated repression of E-cadherin.

### WT1 promotes the epithelialization of renal cells

**Figure 5:**
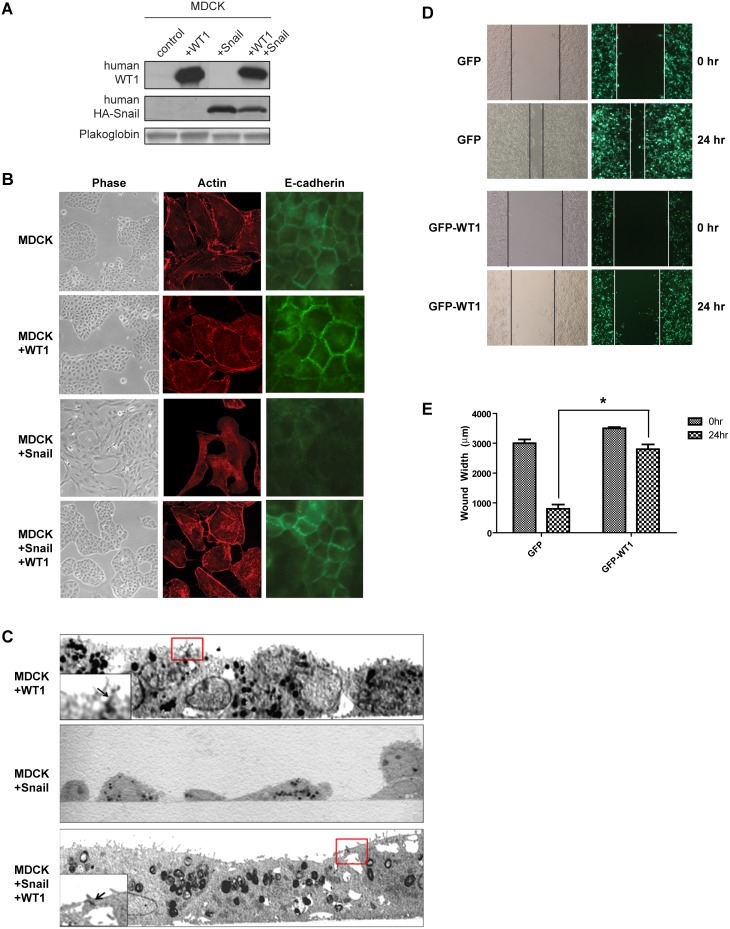
WT1 preserves epithelial junctions and suppresses motility in renal cells. (A) MDCK cells were transfected as indicated and protein expression was assessed by immunoblot. (B) Representative immunofluorescene images of MDCK cells transfected with Snail alone or Snail and WT1. (C) Electron microscopy images of MDCK cells transfected with WT1, Snail, or WT1 and Snail. Red boxes indicate cell-cell contact regions showing intercellular junctions junctions enlarged in the inset (arrow). Note the absence of intercellular contacts and spindle morphology of Snail-transfected cells. (D) HEK293T cells were transfected with either GFP or GFP-WT1 and a scratch motility assay was performed. (E) Quantification of the width of the wound at the indicated time points. Graph depicts mean±SD of three independent experiments. *, *P*<0.05.

To probe the influence of WT1 on cellular phenotype, we analyzed MDCK cells transfected with human WT1 alone, human HA-tagged Snail alone, or both HA-Snail and WT1 (Fig. 5A). Control MDCK and WT1-expressing cells exhibited epithelial morphology, cortical actin arrangement, and abundant E-cadherin expression at cell-cell contacts, whereas overexpression of HA-Snail resulted in mesenchymal morphology, reorganization of actin into stress fibers, and loss of E-cadherin expression (Fig. 5B). Strikingly, overexpression of WT1 in Snail-expressing MDCK cells restored epithelial morphology, reverted cellular actin back into a cortical organization, and reestablished intercellular E-cadherin (Fig. 5B). Transmission electron microscopy further revealed that epithelial junctions were preserved in MDCK cells co-expressing both WT1 and HA-Snail, but not in cells expressing HA-Snail alone (Fig. 5C).

Epithelial cells are less motile than mesenchymal cells. In order to determine whether WT1 suppresses cell motility, we measured the wound closure of HEK293T cells expressing either control GFP or GFP-WT1. After 24 hr, GFP-transfected cells closed 70% of the wound, but GFP-WT1-expressing cells migrated much slower and only closed 20% of the wound (Fig. 5D, E). Altogether, these observations suggest that WT1 may modulate phenotypic plasticity by upregulating E-cadherin and promoting a more epithelialized cellular phenotype.

### VHL-knockdown RCC cells exhibit both epithelial and mesenchymal characteristics

**Figure 6:**
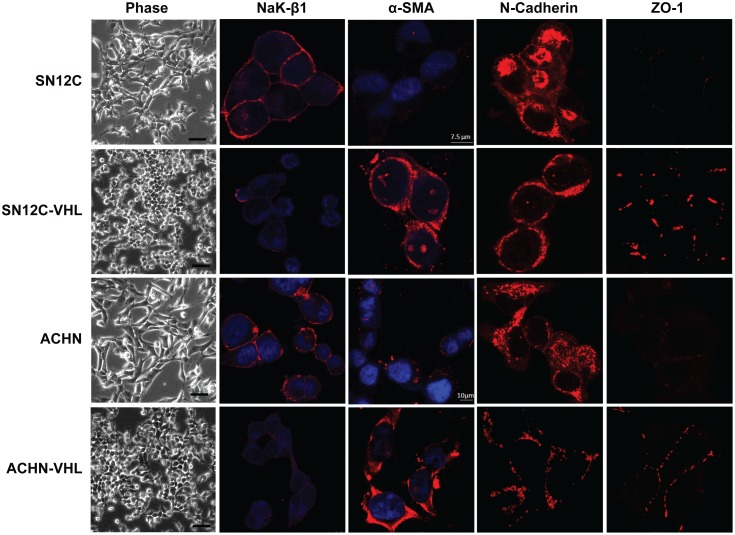
VHL-knockdown alters the expression of EMT markers in RCC cells. Representative phase-contrast and immunofluorescence images of the isogenic SN12C and ACHN cells. Scale bars = 100 µm in the phase-contrast images. Scale bars = 7.5 µm and 10 µm in the immunofluorescence images of the SN12C and ACHN cells, respectively.

Finally, we used the isogenic ccRCC cells to conduct an analysis of cellular morphology and various markers of mesenchymal (α-smooth muscle actin [α-SMA] and N-cadherin, mesenchymal markers upregulated during EMT) vs. epithelial (Na,K-ATPase β_1_ subunit [NaK-β_1_], a cell-cell adhesion molecule downregulated during EMT [31], and zonula occludens-1 [ZO-1], a tight junction marker) differentiation. Morphologically, the parental SN12C and ACHN cells appeared more mesenchymal (Fig. 6). Consistent with the mesenchymal phenotype they expressed higher levels of N-cadherin (in agreement with Fig. 4A) and very low levels of ZO-1 at the cell-cell contact region (Fig. 6). By contrast, these cells expressed high levels of NaK-β_1_ and low levels of α-SMA (Fig. 6). The VHL-knockdown cells exhibited a more clustered, pseudo-epithelial morphology (Fig. 6). These cells displayed reduced N-cadherin (in agreement with Fig. 4A) and markedly enhanced intercellular expression of ZO-1. Strikingly, the expression of NaK-β_1_ was reduced, and α-SMA expression was increased (Fig. 6). These data reveal that although features of both EMT and MET were present in all of these cell lines, the VHL-knockdown cells tended to exhibit a more epithelialized phenotype than the parental cells.

## Discussion

In this study, we demonstrated that WT1 is induced in RCC cells that are deficient in VHL expression. Paradoxically, we observed that WT1 transcriptionally upregulated both Snail, a transcriptional repressor that promotes a mesenchymal phenotype, and E-cadherin, a cell-cell adhesion molecule that maintains an epithelial phenotype. These studies indicate for the first time, to our knowledge, that WT1 can induce what we designate as an epithelial-mesenchymal hybrid transition (EMHT), a differentiation state that includes molecular signatures and morphological features of both MET and EMT in carcinoma cells. We suggest that WT1-induced EMHT is involved in providing phenotypic plasticity to tumor cells.

### VHL deficiency and WT1 expression

Functional inactivation of VHL occurs in the majority of sporadic ccRCCs [22] and leads to constitutive expression of HIF in the absence of hypoxia [32]. We found that knockdown of VHL was associated with increased WT1 expression in ccRCC cells. WT1 has been shown to be a HIF-responsive gene [23], and we found that inhibition of HIF activity with echinomycin resulted in almost complete suppression of WT1 in VHL-deficient cells. Our results are consistent with the findings that VHL deficiency induces WT1 via the stabilization of HIF, however it is likely that additional HIF-independent effects also contribute to the upregulation of WT1 in VHL-deficient cells. Notably, VHL and WT1 exhibit an inverse relationship during kidney development; VHL expression is elevated in the mature renal proximal tubule cells [33] whereas WT1 expression is elevated in the metanephric mesenchyme but decreases as these structures differentiate into the proximal tubules [4], [20]. Since ccRCC is believed to originate from proximal tubule cells [1], our results indicate that the inverse relationship between WT1 and VHL expression during kidney development is recapitulated in renal tumorigenesis.

### Regulation of Snail by WT1

Snail is a transcriptional suppressor that promotes EMT during organ development and cancer progression [15]. The best-characterized function of Snail is the downregulation of E-cadherin expression by binding to specific DNA elements (E-boxes) within the E-cadherin (*CDH1*) promoter [14], [34]. In agreement with Evans *et al.* [25], we found that knockdown of VHL enhanced Snail expression in ccRCC cells, and we have further demonstrated that this occurred *in part* through a WT1-dependent mechanism. Since echinomycin treatment, which abrogates HIF function, did not repress Snail expression completely it is possible that other HIF independent pathways might be involved in the regulation of Snail. WT1 was shown to directly bind to a conserved consensus motif within the *SNAI1* promoter to activate gene expression, and mutation of this site considerably reduced promoter activity. These data are consistent with the study by Martínez-Estrada *et al.*, which reported similar results in a murine model of heart development [5].

### WT1 and E-cadherin expression in ccRCC

E-cadherin is frequently lost or downregulated in many human cancers, including bladder, breast, head and neck, and in various tumor cell lines, and its loss corresponds with EMT and the acquisition of invasiveness [18]. E-cadherin is a target gene of both Snail and WT1, and is repressed by Snail [34], but can be either repressed [5], [28] or upregulated [30] by WT1 depending on the cellular context. Interestingly, we found that expression of WT1 in various renal cell lines resulted in elevated E-cadherin expression despite the observed induction of Snail. These results indicate that Snail is unable to effectively suppress E-cadherin expression in the presence of WT1. Preliminary data indicates that WT1 and Snail binding to the *CDH1* promoter are mutually exclusive, in agreement with the close proximity of the binding sites in this promoter. In the human *CDH1* promoter, the WT1 binding site is located between E-boxes 1 and 2, which is 22 bp downstream from E-box 1 and only 13 bp upstream of E-box 2. Given the proximity of these binding motifs, it is likely that the relative expression of Snail and WT1 may control *CDH1* gene expression. While our results are at variance with the Martínez-Estrada *et al.* study, in which WT1 expression correlated with decreased E-cadherin expression during heart development [5], this difference highlights probable dissimilarities between normal embryological versus cancer development. It is likely that other factors stimulate Snail expression during development to enhance Snail levels and prevent WT1 interaction with the *CDH1* promoter. Of note, while the findings of Evans *et al.* demonstrated that loss of VHL promoted Snail- and SIP1-mediated repression of E-cadherin expression [25], their study utilized the VHL-null ccRCC cell line 786-O which does not express detectible WT1 (our unpublished data). Therefore, it is anticipated that loss of VHL function might bring about different phenotypes based upon the molecular composition of tumor cells and upon other cellular signals that can foster Snail expression. Future investigation will be necessary to understand these various mechanisms.

### Phenotypic Plasticity by WT1

We observed that WT1 expression in renal cells was consistently associated with the gain of epithelial morphology despite the enhanced expression of Snail, a key molecule involved in the induction of mesenchymal characteristics. Overexpression of Snail in MDCK cells induced mesenchymal characteristics as expected, but co-expression with WT1 led to a partial reversion to a more epithelial-like morphology. In cells undergoing EMT, increased stress fibers facilitate cell motility [35]. The presence of WT1 in Snail-overexpressing MDCK cells resulted in reduced stress fibers, and WT1 overexpression attenuated the migration of HEK293T cells. The adherens junction marker E-cadherin was localized to the cell-cell contacts in WT1 and Snail co-expressing cells. Most importantly, electron microscopic images of these cells revealed close intercellular adhesion and epithelial junctions, which are the hallmark of the epithelial phenotype. Furthermore, in the VHL-knockdown ccRCC cells, enhanced E-cadherin and ZO-1 and reduced N-cadherin expression suggests the promotion of epithelial characteristics. Interestingly, these cells also displayed reduced NaK-β_1_ and enhanced smooth muscle actin. The expression patterns of these latter markers are consistent with the acquisition of mesenchymal characteristics. Thus, in VHL-knockdown WT1-expressing cancer cells, markers of both EMT and MET coexisted, and these cells displayed epithelial-like morphology. Taken together, our results indicate that WT1 imparts phenotypic plasticity in normal and cancerous kidney cells.

Carcinoma cells can exhibit a wide spectrum of phenotypes that range from fully epithelial to fully mesenchymal [18]. The phenotype of carcinoma cells is now recognized to be more plastic than previously assumed, and can transition along the epithelial-mesenchymal spectrum depending on the molecular composition of both the cells and the tumor microenvironment. We speculate that WT1 enhances phenotypic plasticity by promoting the expression of both EMT and MET markers, which confers cells with the characteristics of both phenotypes. This in turn may allow tumor cells to more readily switch their differentiation to a mesenchymal phenotype (for invasiveness and metastasis) or an epithelial phenotype (for metastatic colonization/proliferation) [17] depending on the demands of the tumor microenvironment.

## Materials and Methods

### Cell culture

Human clear cell RCC (SN12C, SN12C-VHL, ACHN and ACHN-VHL), embryonic kidney (HEK293T), and canine kidney (MDCK) cells were maintained in Dulbecco's modified Eagle's medium (DMEM) supplemented with 10% fetal bovine serum (FBS), 2 mM L-glutamine, 25 U/mL penicillin, and 25 µg/mL streptomycin. SN12C, SN12C-VHL, ACHN, and ACHN-VHL cells were kindly provided by Dr. Charles Sawyers (Memorial Sloan-Kettering Cancer Center, NY). HEK293T and MDCK cells were purchased from the American Type Culture Collection (Manassas, VA). The generation of MDCK stably transfected with Snail1-HA has been already described [34]. Stable ectopic expression of WT1 was carried out transfecting pREP-WT1 (-KTS) (kindly provided by Dr. G. Gil, IMIM) or pREP4 as control. Transfection was performed in control or Snail1-HA-expressing MDCK cells using Lipofectamine-Plus reagent (Life Technologies, Grand Island, NY) under the manufacturer's specifications; stabley-expressing clones were isolated and selected with hygromycin (0.4 mg/ml, Life Technologies) and WT1 expression was tested by immunoblot.

Transient RNAi transfections using VHL-, WT1-, and Snail-targeting SMARTpool siRNA oligonucleotides or a non-targeting control sequence (Dharmacon, Lafayette, CO) and GFP-WT1 kindly provided by Dr. Gail Fraizer, Ken State University [36], were performed with 500,000 cells, using the Amaxa Nucleofector (Lonza Group, Basel, Switzerland). The siRNA oligos were co-transfected with GFP plasmid to determine efficiency of transfections. After 48 hrs, cells were lysed for protein and mRNA determination.

### Antibodies and Reagents

We purchased primary antibodies specific for WT1, Snail, N-cadherin (Santa Cruz Biotechnology, Santa Cruz, CA), E-cadherin 24E10, GAPDH (Cell Signaling Technology, Danvers, MA), E-cadherin clone 36, plakoglobin, VHL (BD Biosciences, San Jose, California), histone H3 (Upstate BioTech, Lake Placid, NY), rabbit IgG (Life Technologies), anti-HA (Roche Applied Science, Penzberg, Germany), α-SMA (Sigma-Aldrich, St. Louis, MO), ZO-1 (Thermo Fisher Scientific, Waltham, MA), Na,K-ATPase β_1_-subunit (Abcam, Cambridge, United Kingdom). Horseradish peroxidase (HRP)-conjugates secondary antibodies for immunoblot analysis were purchased from Cell Signaling Technology, and FITC- and Texas Red-labeled secondary antibodies for immunofluorescence were obtained from Jackson ImmunoResearch Laboratories (West Grove, PA). Echinomycin was purchased from Sigma-Aldrich (St. Louis, MO).

### Immunoblot Analysis

Immunoblotting was performed as outlined previously [37]. Briefly, whole cell lysates were prepared in lysis buffer (95 mM NaCl, 25 mM Tris, pH 7.4,0.5 mM EDTA, 2% SDS, 1 mM PMSF and protease inhibitors). Lysates were sonicated and cleared of cell debris by centrifugation. The supernatants were used for protein determination (DC protein assay, Bio-Rad, Hercules, CA) and equal amounts of protein were separated by SDS-PAGE, transferred to nitrocellulose membrane and subjected to immunoblot analysis with primary antibodies. Images were generated using Photoshop (Adobe Systems Inc., San Jose, CA) and relative quantification was performed using ImageJ (NIH, Bethesda, MD).

### Quantitative Real-time PCR

Total RNA was prepared from cell using the RNA Aqueous Kit (Ambion). Gene expression was quantified by reverse-transcription using the High Capacity cDNA Synthesis Kit (Applied Biosystems) followed by real-time quantitative PCR using gene expression assays purchased from Applied Biosystems. DNA amplifications were performed according to manufacturer's instructions. Samples were assayed in a 384-well format in triplicate using a 7900HT Fast Real-Time PCR system (Applied Biosystems, Foster City, CA). Variation in cDNA loading was normalized to 18S rRNA expression and relative expression was determined using the ΔΔCt method of relative quantification (RQ). Graphs represent the average RQ value with error bars (standard error of the RQ value) from one of three independent experiments.

### Chromatin Immunoprecipitation (CHIP)

2×10^6^ cells were cross-linked with 1% formaldehyde at 37°C for 15 min, lysed (1% SDS, 10 mmol/L EDTA, 50 mol/L Tris-HCl, pH 8.1) and chromatin was sonicated to fragments of about ∼200–1,000 bp. Immunoprecipitations were done using anti-WT1 antibody, anti-rabbit IgG as negative control, and anti-acetyl-histone H3 antibody as positive control. DNA was extracted, purified, and amplified by PCR using primers against Snail1 promoter, (forward) 5′-CGAAAGGCCTTCAACTGCAAATACTGC-3′ and (reverse) 5′-TGTGTGGCTTCGGATGTGCATCTT-3′. Amplification products were visualized on 2% agarose gels.

### Luciferase Assays

Construction of the plasmid containing the human Snail and E-cadherin promoter fused to the luciferase reporter gene has previously been described [16], [34]. Mutagenesis of the WT1 binding site was performed using the Quick Change Multiple Site-Directed Mutagenesis Kit (Strategene) following the manufacturer's protocol, using the forward primer 5′-CAAGCCCGAGGCGGAAACGGGCGTCGGAAGG-3′); and the reverse primer 5′-CCTTCCGACGCCCGTTTCCGCCTCGGGCTTG-3′. The introduction of the desired mutation was verified by sequencing analysis.

To measure promoter activity, cells were co-transfected with plasmids containing either the Snail or E-cadherin promoter. The cell extracts were prepared and reporter gene activity was determined using the Dual-Luciferase Reporter Assay (Promega Corporation, Madison, WI) [37]. The Victor plate reader was used to measure luciferase reporter units (PerkinElmer, Waltham, MA). Luciferase values were normalized to *Renilla* reporter activity.

### Immunofluorescence Microscopy

Cells were seeded on poly-L-Lysine coated glass coverslips placed in 12-well plates. The cells were grown overnight before processing them for immunoflourescence. The cells were rinsed with PBS containing MgC1_2_ (1 mM) and CaCI_2_ (0.1 mM) (PBS-CM) and fixed with cold methanol for 20 min at −20°C. The cells were then rehydrated with PBS-CM containing 0.2% BSA (PBS-CM-BSA), washing three times for 10 min each. Subsequently, the cells were incubated with the primary antibodies (1∶200 dilution in PBS-CM-BSA) for 1 hr, washed three times for 10 min in PBS-CM-BSA, and then incubated for 30 min at room temperature with secondary antibodies (1∶200 dilution in PBS-CM-BSA). TO-PRO-3 (Thermo Fisher Scientific) was used for nuclear staining at a dilution of 1∶500. Actin fibers were visualized by incubating permeabilized cells with Rhodamine-labelled phalloidin (Thermo Fisher Scientific). After secondary labeling the coverslips were again rinsed with PBS-CM-BSA three times for 10 min each. The coverslips were then mounted in SlowFade Gold Anti-Fade Reagent (Thermo Fisher Scientific). Phase-contrast images were obtained using a Leica DM IL microscope equipped with a DFC420 camera and Leica Application Suite Software (Leica Microsystems GmbH, Wetzlar, Germany), and fluorescence images were captured using a Leica TCS SP5 scanning confocal microscope (Leica Microsystems Inc., Buffalo Grove, IL) using the 63× oil objective lens. Images were processed using Photoshop (Adobe Systems Inc., San Jose, CA).

### Scratch Wound Migration Assay

HEK293T cells were transfected with GFP or GFP-WT1 as described above. Cells were grown to confluence in 6-well dishes and a wound was made using a pipette tip. Cells were washed with PBS and fresh media was added to each plate. Dishes were visualized at 40× magnification and photographed at t = 0 and 24 hr after the wound was made using a Leica DM IL microscope equipped with a DCF 420 C camera and Leica Application Suite Software (Leica Microsystems GmbH, Wetzlar, Germany). Images were processed using Photoshop (Adobe Systems Inc., San Jose, CA). Wound coverage was reported by measuring the distance between the scratch borders at t = 0 hr and t = 24 hr.

### Tissue Sectioning and Electron Microscopy

Ten days-post confluent cells were fixed with glutaraldehyde (2.5%) for 30 minutes and post-fixed in OsO_4_ (2%) for 1 min at room temperature in light-protected chambers. Cells were washed in dH_2_O and dehydrated by washing in different concentrations of ethanol (from 30 to 100%). Samples were then incubated in N-(2-hydroxypropyl)methacrylamide (HPMA) (Merck Chemicals, Darmstadt, Germany) (twice, for 15 minutes) and included in tEpon-812 (Tousimis Research, Rockville, MD). The different samples were additionally incubated for 18 hours at 37°C and 24 hours at 60%C in Epon. Ultrathin sections were obtained in a LKB ultra-microtome, stained with uranyl acetate and lead citrate, and analyzed in Philips CM100 electronic microscope at 5,000X.

### Statistical Analysis

All statistical analyses used the two-tailed unpaired Student *t* test and were calculated using GraphPad Prism software (GraphPad Software Inc., La Jolla CA). Significance was defined as *P*<0.05 and is denoted by asterisks in the figures.
